# Supplementary material for: Grapevine Plants Management Using Natural Extracts and Phytosynthesized Silver Nanoparticles
Source: Materials (Basel). 2022 Nov 18;15(22):8188. doi: 10.3390/ma15228188 (PMC9697161; doi:10.3390/ma15228188)
Supplement: Supplementary file 1 [file materials-15-08188-s001.zip › materials-2043122-supplementary.pdf]

Supplementary Material

Grapevine plants management using natural extracts and phytosynthesized silver nanoparticles

Diana Elena Vizitiu[1], Daniela Ionela Sardarescu[1,2*], Irina Fierascu[3,4], Radu Claudiu Fierascu[2,3], Liliana Cristina Soare[5], Camelia Ungureanu[2*], Elena Cocuta Buciumeanu[1], Ionela Catalina Guta[1], Letitia Mariana Pandelea[1]

[1] National Research and Development Institute for Biotechnology in Horticulture Stefanesti, 37 Bucharest—Pitesti Str., 117715 Argeș, Romania
[2] Faculty of Chemical Engineering and Biotechnologies, University "Politehnica" of Bucharest, Bucharest, 313 Splaiul Independentei Str., 060042 Bucharest, Romania
[3] The National Institute for Research & Development in Chemistry and Petrochemistry, ICECHIM, 202 Spl. Independentei, 060021 Bucharest, Romania
[4] Faculty of Horticulture, University of Agronomic Sciences and Veterinary Medicine of Bucharest, 011464 Bucharest, Romania
[5] Natural Science Department, Faculty of Sciences, Physical Education and Informatics, University of Pitesti, 1 Targul din Vale Str., 110040 Pitesti, Romania
* Correspondence: ionela.toma93@yahoo.com (D.I.S.); ungureanucamelia@gmail.com (C.U.)

1. Experimental procedure

1.1. Obtaining and Characterization of Vegetal Extract and Silver Nanoparticles

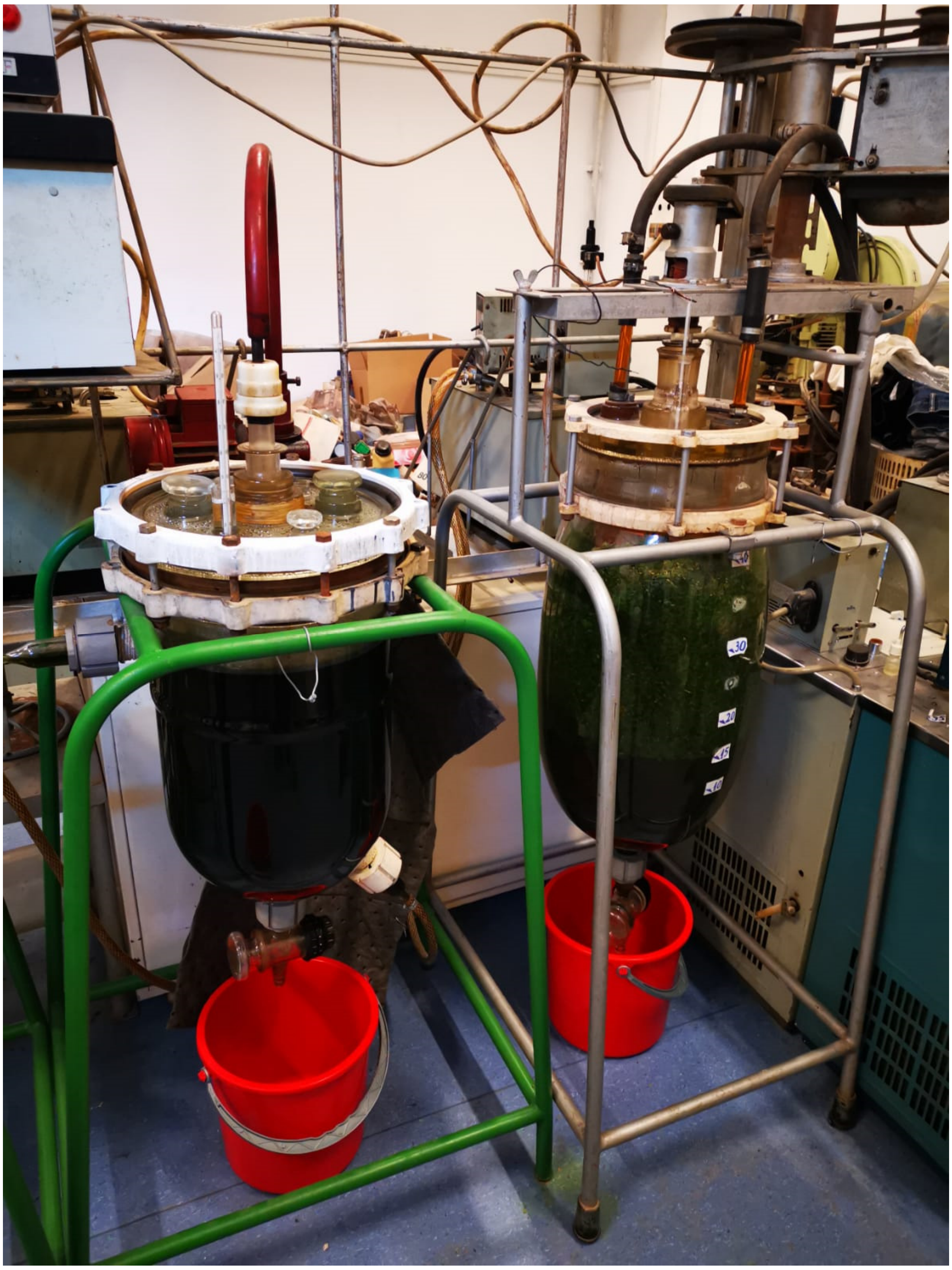

Figure S1. Obtaining the natural extracts in large scale reactors.

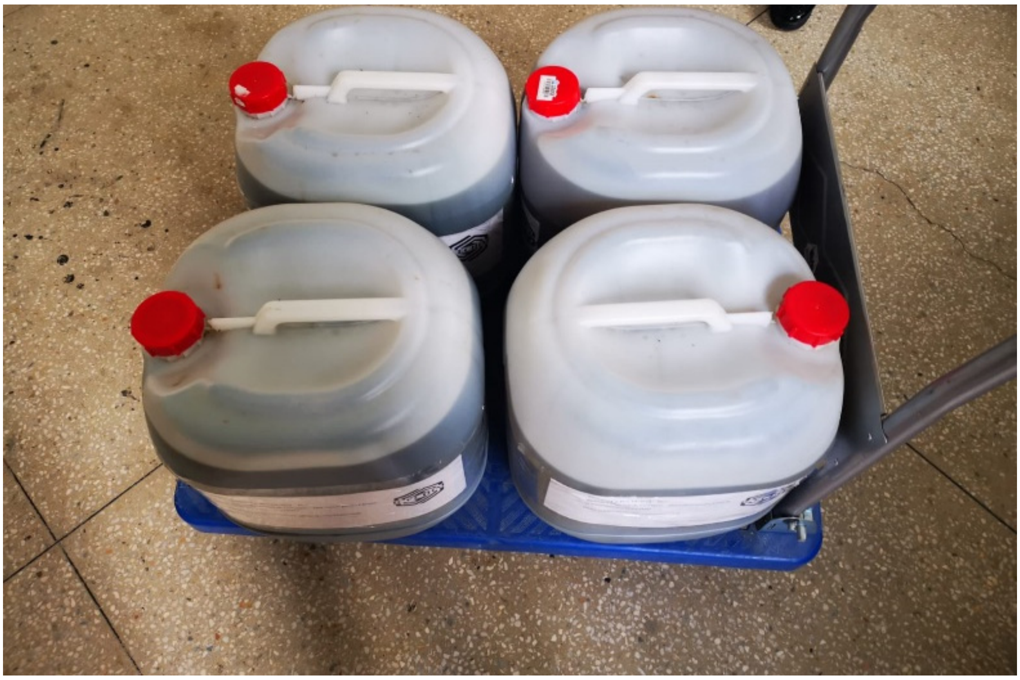

Figure S2. The two types of recipes prepared for field application.

1.2. Evaluation of Total Phenolic Content of the Extracts

For the determination of the total phenolic content was used a method previously presented [1]. Briefly, 10% diluted Folin-Ciocalteu reagent (150 μL) and 0.7 M Na_2CO_3 (120 μL) were added to the extracts (30 μL) and following their incubation for 30 min with shaking at room temperature, the absorbance was spectrophotometrically measured at 765 nm and the results were expressed as milligrams of gallic acid equivalents (GAE) per gram of extract using the following equation:

$$C_{TP} = c \times \frac{V}{m}$$

where: C_{TP}—total phenolic content (mg/g) in GAE (gallic acid) equivalent, C—Concentration of gallic acid obtained from calibration curve in mg/mL, V- volume of extract in mL, m—mass of extract in grams.

The calibration curves were constructed using analytic standards gallic acid (SigmaAldrich, Germany). The experiments were carried out in triplicate.

1.3. Field Experiments

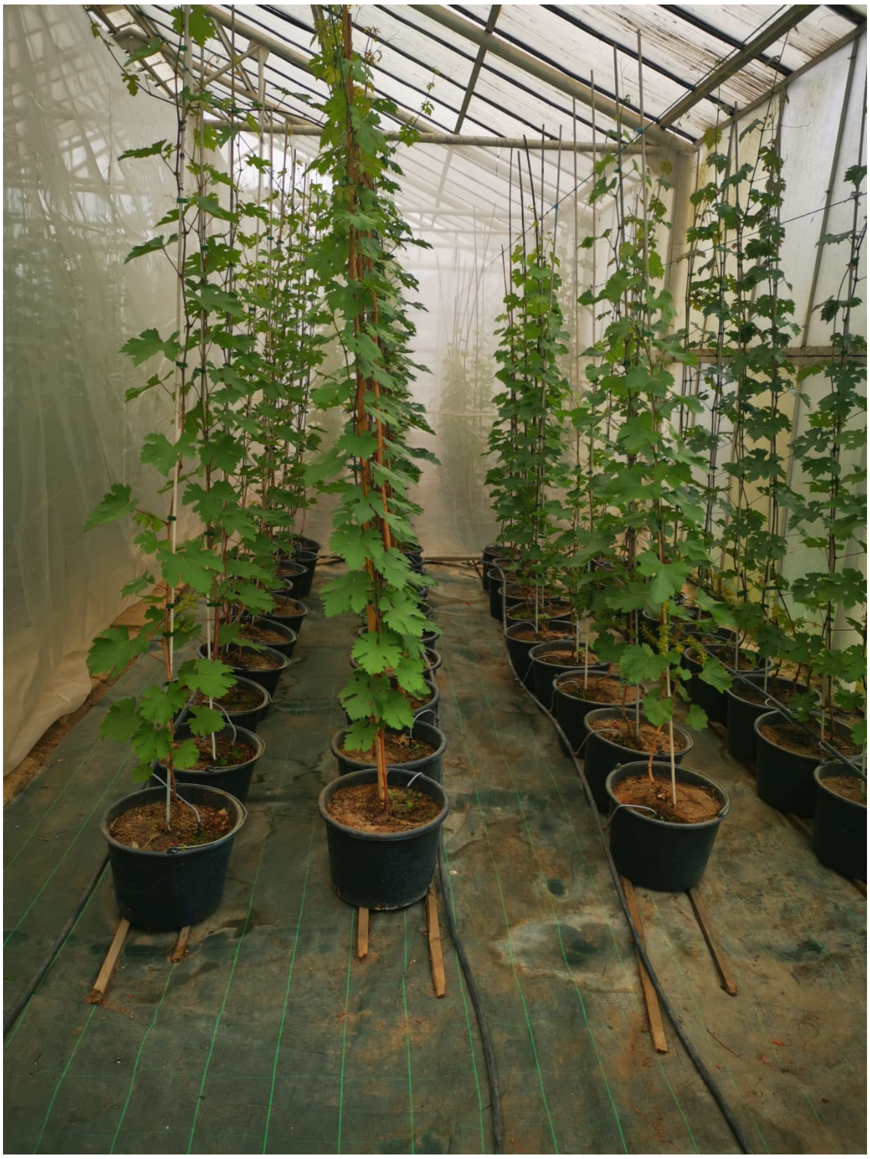

Figure S3. Aspects of the greenhouse experiments.
